# Editorial for the Special Issue “Advances in Plant–Soil–Microbe Interactions”

**DOI:** 10.3390/microorganisms14092017

**Published:** 2026-09-11

**Authors:** Lily X. Zelaya-Molina, Sergio de los Santos-Villalobos

**Affiliations:** 1 Centro Nacional de Recursos Genéticos-INIFAP, Tepatitlán de Morelos 47600, Jalisco, Mexico; 2 Departamento de Ciencias Agronómicas y Veterinarias, Instituto Tecnológico de Sonora, Ciudad Obregón 85000, Sonora, Mexico

Plant–soil–microbe interactions are central to terrestrial ecosystems and are important for agriculture and environmental management. Plants interact with diverse microbial communities in the rhizosphere and endosphere, and these interactions are affected by soil properties, agricultural practices, plant characteristics, and environmental conditions. Changes in these factors can alter microbial community composition and activity, affecting nutrient cycling, plant development, and plant responses to environmental stress. This Special Issue, titled “Advances in Plant–Soil–Microbe Interactions”, includes 18 manuscripts that examine these interactions at different levels, from changes in soil microbial communities under different environmental and agricultural conditions to the characterization of bacteria with plant growth-promoting traits and the use of microbial consortia for crop production. Together, the contributions provide different perspectives on the relationships among plants, soils, and microorganisms and their importance for both natural and managed ecosystems.

Environmental conditions are an important factor in the composition and activity of plant-associated microbial communities. Several contributions in this Special Issue examine microbial responses to environmental conditions; for example, two studies focus on saline environments and provide complementary views of bacterial adaptation to these conditions. Research on *Aeluropus sinensis* evaluated bacterial communities in the rhizosphere and endorhizosphere across saline lands, while another study isolated rhizosphere-associated bacteria from saltgrass (*Distichlis spicata*) and evaluated their ability to tolerate saline conditions and promote plant growth [1,2]. Water availability was also considered in a study of a jujube/cotton intercropping system, where different irrigation rates were associated with changes in soil bacterial diversity and lint yield [3]. These studies show that microbial communities respond to the conditions in which plants grow and that factors such as salinity and water availability can affect both microbial communities and plant performance. This is particularly relevant in agricultural systems where environmental stress can limit crop production and where microorganisms adapted to specific conditions may have value as part of strategies to support plant growth.

Plant characteristics and agricultural management also have an important role in soil microbial communities. Root exudates can influence microbial activity and the fate of carbon in soil, as shown by the study of microbial necromass formation and soil organic carbon stabilization under long-term nitrogen fertilization [4]. Crop management can have similar effects. The study of different crop rotations in *Brassica napus* evaluated changes in microbial diversity and enzyme activities in rhizosphere soil under different cropping sequences [5]. Other contributions considered changes in microbial communities after changes in vegetation. The study of *Idesia polycarpa* followed changes in soil microorganisms after the establishment of a new plantation, while research on *Larix olgensis* showed relationships between tree species mixtures, microbial network complexity, and soil nutrient cycling [6,7]. Although these studies involve different plant species and ecosystems, they point to the role of plants and management practices in determining soil microbial communities and their functions. They also show that changes in microbial communities can be related to processes that are important for soil quality and nutrient availability.

A second group of studies focuses on individual microorganisms with potential benefits for plants. The characterization of bacterial isolates from agricultural systems provides information on microorganisms that combine several traits related to plant growth and plant health. *Bacillus velezensis* TRQ67, isolated from wheat rhizosphere soil in the Yaqui Valley, Mexico, was evaluated for its ability to promote wheat growth and inhibit fungal phytopathogens [8]. Another study characterized the draft genome of *Bacillus* sp. strain 11B20, a bacterium associated with maize in the same region, and described genomic and functional characteristics related to its plant-growth-promoting potential [9]. The Special Issue also includes research on IAA-producing endophytic *Bacillus* spp. and their effects on barley growth, as well as rhizosphere bacteria from saltgrass with saline tolerance and plant-growth-promoting traits [2,10]. These studies show the range of functions found among plant-associated bacteria and the potential of selected strains for agricultural use. They also support the use of functional assays together with molecular and genomic approaches when selecting microorganisms for further evaluation.

The use of microbial consortia is another important topic in this Special Issue. The study of SynCom-SASW01 in wheat evaluated how a synthetic microbial community affects rhizosphere–endophytic interactions and drought resistance [11]. Microbial consortia may offer advantages over individual strains because different microorganisms can contribute complementary functions. However, the performance of a consortium also depends on the interactions among its members and on the environmental conditions in which it is applied. For this reason, the selection of compatible microorganisms and the evaluation of their behavior under conditions that are relevant to agriculture are important steps toward the development of effective microbial inoculants. This approach adds another level to the study of plant–microbe interactions, from the characterization of individual strains to the design of microbial communities with defined functions.

Taken together, the contributions to this Special Issue show the wide range of processes involved in plant–soil–microbe interactions, from changes in microbial communities under different environmental and agricultural conditions to the selection of microorganisms with traits of interest for crop production. The studies also illustrate how results obtained at different levels, from soil microbial communities to individual strains and microbial consortia, can complement each other and provide a more complete view of these systems. Community-level studies help describe changes in microbial diversity and structure, whereas functional and genomic approaches provide information on microbial traits and their possible roles in plant performance. Studies with microbial consortia add another perspective by considering interactions among microorganisms and their effects on plants. Together, these approaches can help identify microorganisms and microbial communities with potential applications while also providing information on the conditions that may affect their performance.

Further work will be needed to determine how the results obtained under controlled or specific experimental conditions translate to different soils, crops, and environments. Field studies, together with physiological, ecological, and genomic approaches, can help clarify the consistency of microbial effects and the factors that determine their success. This is especially important for developing microbial products for agriculture, where results can vary with soil properties, climate, crop genotype, and management practices. A better connection between laboratory studies, field evaluation, and the ecological context of microbial communities may therefore help move promising microorganisms from experimental systems toward practical applications in crop production and soil management. We hope that this Special Issue, titled “Advances in Plant–Soil–Microbe Interactions”, will contribute to current research in this field and encourage further work on the complex relationships among plants, soils, and microorganisms. The studies presented here cover different environments, crops, microbial groups, and experimental approaches, but all contribute to a better view of the role of microorganisms in plant performance and soil processes. Continued research in this area may support the development of microbial-based strategies for sustainable crop production, soil restoration, and the management of environmental stress.
